# Monitoring the Growth of a Microbubble Generated Photothermally onto an Optical Fiber by Means Fabry–Perot Interferometry

**DOI:** 10.3390/s21020628

**Published:** 2021-01-18

**Authors:** J. Gabriel Ortega-Mendoza, Placido Zaca-Morán, J. Pablo Padilla-Martínez, Josué E. Muñoz-Pérez, José Luis Cruz, Miguel V. Andrés

**Affiliations:** 1 División de Posgrado, Universidad Politécnica de Tulancingo, Tulancingo de Bravo, Hidalgo C.P. 43629, Mexico; josue.munos@upt.edu.mx; 2 Instituto de Ciencias, Benemérita Universidad Autónoma de Puebla, Ecocampus Valsequillo, Puebla C.P. 72960, Mexico; placido.zaca@correo.buap.mx (P.Z.-M.); juan.padilla@correo.buap.mx (J.P.P.-M.); 3 Departamento de Física Aplicada y Electromagnetismo, Universidad de Valencia, Dr. Moliner 50, 46100 Burjassot, Spain; jose.l.cruz@uv.es (J.L.C.); miguel.andres@uv.es (M.V.A.)

**Keywords:** microbubble, Fabry–Perot, optical fiber, cavity, vibrometer

## Abstract

In the present paper, we show the experimental measurement of the growth of a microbubble created on the tip of a single mode optical fiber, in which zinc nanoparticles were photodeposited on its core by using a single laser source to carry out both the generation of the microbubble by photothermal effect and the monitoring of the microbubble diameter. The photodeposition technique, as well as the formation of the microbubble, was carried out by using a single-mode pigtailed laser diode with emission at a wavelength of 658 nm. The microbubble’s growth was analyzed in the time domain by the analysis of the Fabry–Perot cavity, whose diameter was calculated with the number of interference fringes visualized in an oscilloscope. The results obtained with this technique were compared with images obtained from a CCD camera, in order to verify the diameter of the microbubble. Therefore, by counting the interference fringes, it was possible to quantify the temporal evolution of the microbubble. As a practical demonstration, we proposed a vibrometer sensor using microbubbles with sizes of 83 and 175 µm as a Fabry–Perot cavity; through the time period of a full oscillation cycle of an interferogram observed in the oscilloscope, it was possible to know the frequency vibration (500 and 1500 Hz) for a cuvette where the microbubble was created.

## 1. Introduction

Fabry–Perot sensing devices have been widely used in several applications, such as hyperspectral images [1], autocorrelation of ultra-short pulse signals [2], medical applications [3], and biosensors [4,5], among others. Fabry–Perot (FP) sensors based on optical fibers offer advantages over electronic sensors; for example, they are safe, free from electromagnetic interference, compact [6,7,8], and have a high sensitivity [9,10,11]. Nowadays, interferometric optical fiber sensors have attracted broad interest for their applications in sensing temperature, refractive index, strain measurement, pressure, acoustic waves, vibration, magnetic field, and voltage. Most of them are studied by generating a microbubble on the tip of an optical fiber, which is considered to be a resonant cavity formed by the vapor–liquid interface and the fiber tip [12,13,14,15,16,17].

Recently, Chen-Li Zhang et al. [18,19] reported the generation of two kinds of optical fiber Fabry–Perot sensors by using a gas microbubble to measure temperature and sucrose concentration. In the first sensor [18], a gold nanofilm was deposited on the tip of the optical fiber, which was submerged in water (absorption coefficient of 10.9 cm^−1^) in order to generate a microbubble, using a laser source at 1550 nm. However, an optical power greater than 50 mW was necessary in order to induce the microbubble and implement the sensor. In the second sensor [19], carbon nanotubes were deposited on the optical fiber end, and two laser sources at 980 and 1500 nm were required. The first source was used to generate a microbubble, and the second source was used to monitor the microbubble’s size. In both studies, the authors worked with the interference fringes in the wavelength domain, which were visualized with help of an Optical Spectrum Analyzer (OSA). It is important to note that the interferometry to determine the size of a microbubble has advantages compared to image analysis, since the interferometry is distinguished by its high resolution, speed of the measurement, simplification, and enhanced robustness of the setup, among other things. Image analysis to obtain the size of a microbubble can generate errors, due to an image out of focus, the resolution of the camera, the inclination and symmetry of the bubble, etc.

In this paper, we present the experimental measurement of the growth of a microbubble created on the tip of an optical fiber with ZnNPs photodeposited on its core. In this work, we used a single source of laser light to carry out the photodeposition, the generation of the microbubble and the characterization of its size in the time domain. The microbubble is considered to be an Fabry–Perot cavity whose temporal evolution and diameter are quantified on account of the number of interference fringes (NIF) obtained by using a photodetector connected to an oscilloscope. It is important to mention that working in the time domain is simpler than in the wavelength domain, mainly for the following two reasons: i) it is possible to use a monochromatic source, and ii) it is not necessary to use sophisticated equipment like an OSA to visualize the interference fringes; only requires a detector and a microcontroller that graphs the detector signals or an oscilloscope. The results obtained by using interferometry were compared with images obtained by a CCD camera, in order to verify the diameter of the microbubble. The experimental results using the microbubble as vibrometer sensor showed that it is possible to know the vibration frequency for the cuvette, as well as the displacement of the microbubble’s surface. In our case, the microbubble was used to detect oscillations in the frequency range 500–1500 Hz

## 2. Operation Principle

**Figure 1:**
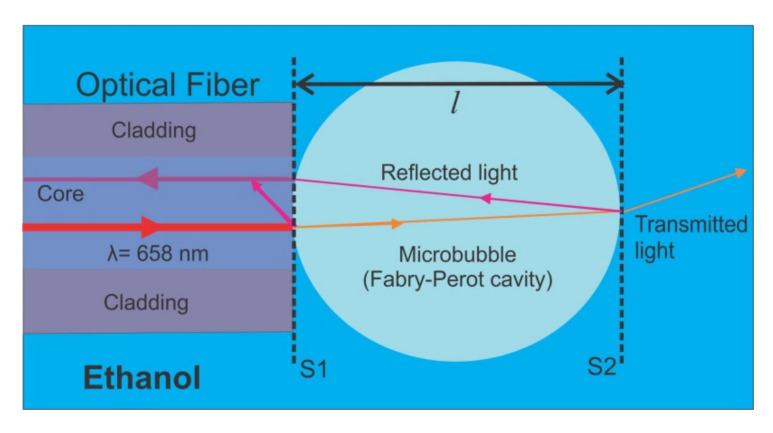
Diagram of the interaction of light into a microbubble, which is considered as a Fabry–Perot cavity.

The study of the size of small objects by means of a Fabry–Perot interferometer has been very important in optical applications. In this work, the analysis of this type of interferometers is used as an alternative method to monitor and quantify the growth of microbubbles induced at the tip of an optical fiber. One of the main advantages of this technique, as compared to the typical methods used (images), is the high-resolution power (nanometers), because the same structure of the bubble is considered as a resonant cavity for the laser, thus making this configuration simple and less robust experimentally. In this section, the principle of operation of the Fabry–Perot interferometer is described as an alternative method to monitor and quantify the diameter of the microbubble. Figure 1 shows a microbubble at the tip of an optical fiber. In this image, it is possible to see its analogy with a Fabry–Perot interferometer by considering the S1 and S2 surfaces as semi-mirrors and analyzing the rays reflected from them. Considering a monochromatic light source, the Fabry–Perot interferometer allows us to measure the diameter with high resolution by counting the interferometric fringes, having in mind that a constructive interference is produced when the following condition is fulfilled:

(1) 2nlcosθ=mλ

where n is the refractive index of the medium through which light travels (*n* = 1 for a microbubble vapor), *l* is the size of the cavity, m is an integer, and *λ* and *θ* are the wavelength and the angle at which the cavity is illuminated, respectively (with the normal to the reflective surfaces). If we consider that the ray is perpendicular to the reflecting surfaces (*θ* = 0°), we obtain the following:

(2) $$l=\frac{m\lambda }{2}$$

A more accurate model can be developed by taking into account a Gaussian beam approximation for the diffraction of the fundamental mode in the bubble [20]. Such a model could describe properly the amplitude, as well as the phase, of the signal reflected and coupled back into the fiber. However, because our measurements are based on counting the interferometric fringes, the amplitude is not relevant—provided it is big enough to be measured—and the error of our ray model is limited to a fraction of a fringe in accordance to the maximum value of the Gouy phase shift that our model neglects.

From Equation (2), it is possible to know the size of the Fabry–Perot cavity by counting the maximum number of interference fringes displayed in the oscilloscope, with the help of a photodetector. It is well-known that one fringe is equivalent to a change in the optical path difference of one wavelength. In this case, when there are changes of pressure in the liquid, the microbubble undergoes a compression or expansion. If this change of pressure is periodic, the surface S2 (see Figure 1) has a behavior similar to that of a mirror moving with velocity uniform via the Doppler effect. Therefore, the Doppler frequency shift *f_D_* can be written as follows:

(3) $$f_{D}=\frac{2v}{\lambda }$$

where v is the velocity component of the surface S2 in the direction of the beam.

## 3. Experimental Setup

**Figure 2:**
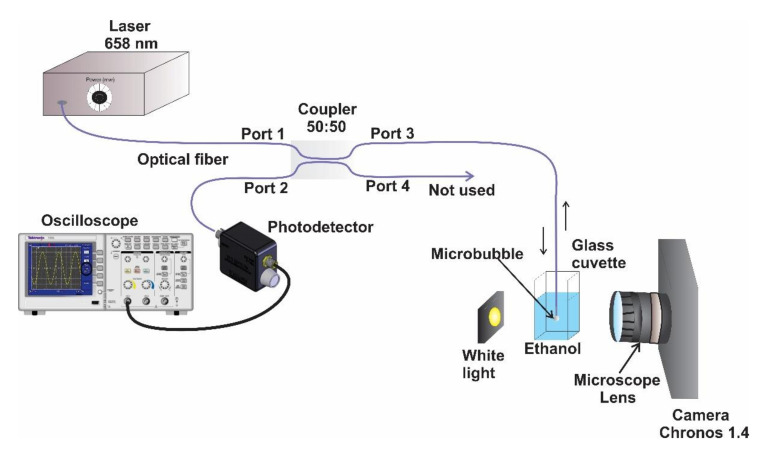
Experimental setup for immobilizing zinc nanoparticles on the tip of an optical fiber and for obtaining microbubble images and interference fringes.

The technique known as photodeposition was used to immobilize ZnNPs on the optical fiber end [21,22,23,24,25]. In this technique, a source of continuous wave radiation is propagated through a single-mode optical fiber (125/9 µm), where the tip of the fiber is immersed in a solution of 4.5 mL of isopropyl alcohol with 10 mg of zinc powder (previously ultrasonified). Furthermore, this experimental setup was used for the formation and characterization of a microbubble created at the tip of the optical fiber. The source light consisted of a single-mode pigtailed laser diode with emission at a wavelength of 658 nm (Mod. LP660–SF60 from Thorlabs Inc., Newton, NJ, USA) that was coupled to a 1 × 2 single mode fiber coupler with a split ration of 50:50 (Figure 2). The incoming laser light was transmitted from Port 1 to Port 3; the end tip of Port 3 was submerged into the solution with nanoparticles. Here, the nanoparticles began to photodeposit on the tip of the optical fiber, and once a 3 dB optical loss was registered, the cuvette with the solution/nanoparticles was changed by another without nanoparticles (only alcohol). Due to the high absorption coefficient of the ZnNPs at the operating wavelength, the light was strongly absorbed by the nanoparticles, heating the solution until a microbubble was formed [21,22,25]. To record the formation and evolution of the microbubble, the cuvette was illuminated perpendicularly to the optical fiber, using a white light source to project the bubble’s shadow on a high-speed camera (Chronos 1.4 from Kron Technologies Inc., Brunaby, BC, Canada), with the help of a microscope lens. Subsequently, the microbubble’s evolution was analyzed in two different ways. In the first case, photographs obtained by the camera were used to measure the size of the bubble, where the fiber cladding diameter was considered as our reference (125 μm). In the second case, the evolution of the bubble was quantified by considering it as a Fabry–Perot cavity, where the fraction of laser light reflected at the vapor–solution interface was transmitted back to Port 3 and exited through Port 2. Finally, the reflected light was measured by a photodetector (Mod. DET025AFC/M from Thorlabs Inc., Newton, NJ, USA, with a bandwidth of 2 GHz) which was connected to an oscilloscope (Figure 2). In this case, the diameter of the microbubble was calculated by counting the number of interference fringes displayed in the oscilloscope.

## 4. Results and Discussions

**Figure 3:**
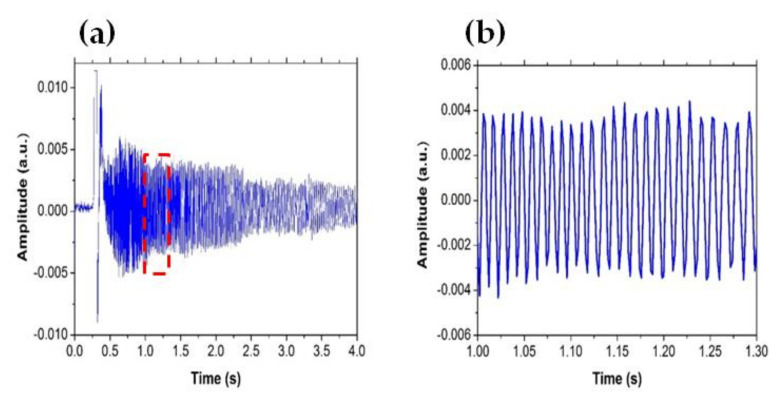
(**a**) Interference fringes visualized with the oscilloscope and (**b**) detail of the marked part of the interferogram.

**Figure 4:**
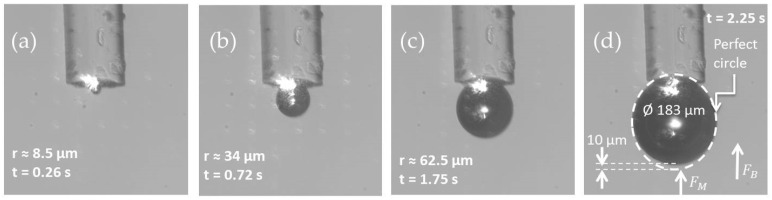
Snapshots show the growth of a microbubble onto optical fiber end over time, under a laser power of 12 mW. Microbubble in (**a**) t = 0.26 s, (**b**) t = 0.72 s, (**c**) t = 1.75 s, and (**d**) t = 2.25 s.

**Figure 5:**
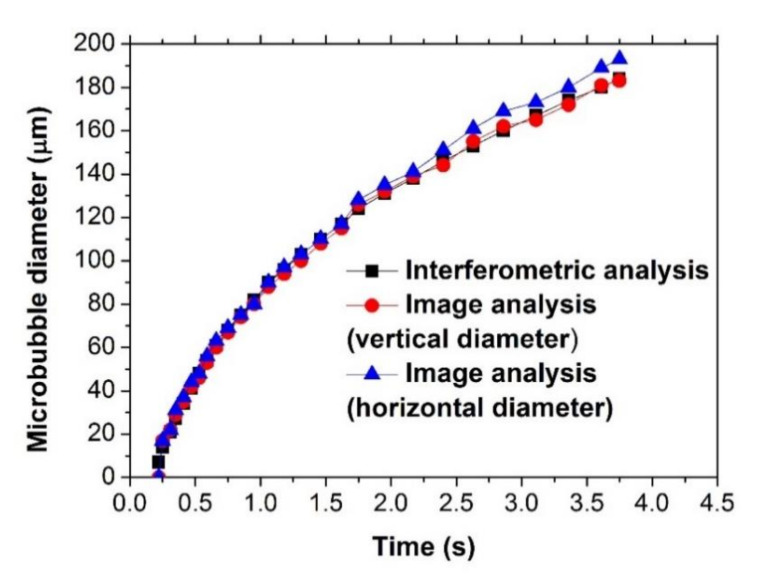
Temporal evolution of the microbubble size.

The reflected signal obtained by the Fabry–Perot cavity when a microbubble is formed on the tip of the fiber is shown in Figure 3. Figure 3 shows the interference fringes during the temporal evolution of the microbubble for 4 s observed in the oscilloscope; a zoomed-in view shows 26 interference fringes in a time interval of 0.3 s. Therefore, the bubble’s diameter can be calculated by the number of interference fringes obtained by the oscilloscope. In this time (4 s), it was possible to count 240 interference fringes, and by taking into consideration Equation (2), the maximum diameter calculated for the microbubble is approximately 75.84 µm. The measuring speed of this method is limited by the response time of the photodetector. In principle, the resolution of the system is 316 nm/fringe; therefore, the minimum diameter of microbubbles that can be measured is 316 nm. However, it should be possible to implement techniques for measuring a fraction of a fringe in order to improve the resolution. Initially, the bubble grows very fast, with interference fringes of high frequency emerging. However, as time goes by, the bubble grows slower (see Figure 4 and Figure 5), causing the interference fringes to be of lower frequency. It is important to note that the amplitude of the interference fringes decreases over time and the period increases. We believe that this behavior is due to divergence of the light beam emitted the fiber: the amount of light that reaches the fiber reflected from surface S2 decreases as the bubble grows. The interference fringes can be visualized in the oscilloscope only when the microbubble grows with a speed greater than 4.75 mm/s; on the contrary, the changes of voltage in the sensor are very slow, and it is not possible to visualize the interference fringes in the oscilloscope. The threshold power for the formation of a microbubble is approximately 4 mW, in order to obtain an optical loss of 3 dB caused by the absorption of light by the zinc nanoparticles.

Additional experimental measurements were carried out to evaluate the dimensions of the microbubble obtained by the interference analysis. Figure 4 shows the tracking of a microbubble generated on the tip of an optical fiber (with ZnNPs photodeposited on its tip) submerged in ethanol over time. Figure 4a–d shows the formation and expansion of the microbubble. In Figure 4d, we can see a white dotted circle around the bubble, which has a diameter of ~183 µm. Here, it is possible to observe that the bubble is not completely spherical, i.e., it has slight deformation in the lower part, which could be due to the convective currents, buoyancy force (FB), or Marangoni force (FM), whose direction is indicated in this figure.

Figure 5 shows the typical temporal evolution of a single microbubble created with 12 mW of laser power inside the solution, using both image analysis and interferometry. By using Equation (2), it was possible to calculate the microbubble’s diameter from interference fringes. In this figure, it is possible to observe that, in the time interval from 0 to 1.5 s, the diameter of the bubble overlaps very well for both analyses (interferometric and imaging); however, it should be noted that, for the analysis of images, it is necessary to use a high-speed camera, which is more expensive than the interferometric setup to achieve similar results. It is necessary to mention that, because the microbubble is not completely spherical, the bubble’s diameter was measured both horizontally and vertically. Here, the circles and triangles represent experimental data obtained by the CCD from the horizontal and vertical diameter of the bubble, respectively, and the squares represent experimental data obtained by the interference fringes, using Equation (2). In this figure, it is possible to observe that the vertical diameter of the microbubble is slightly less than the horizontal diameter; however, the vertical diameter agrees well with the diameter of the microbubble obtained with interferometry analysis, since the vertical axis of the bubble is parallel to the direction of propagation of the laser light inside the bubble (Fabry–Perot cavity). Therefore, from this figure, the non-sphericity of the microbubble is verified. It is important to mention that the image of the microbubble must be well focused in order to obtain a clear image and reduce errors in the measurement.

**Figure 6:**
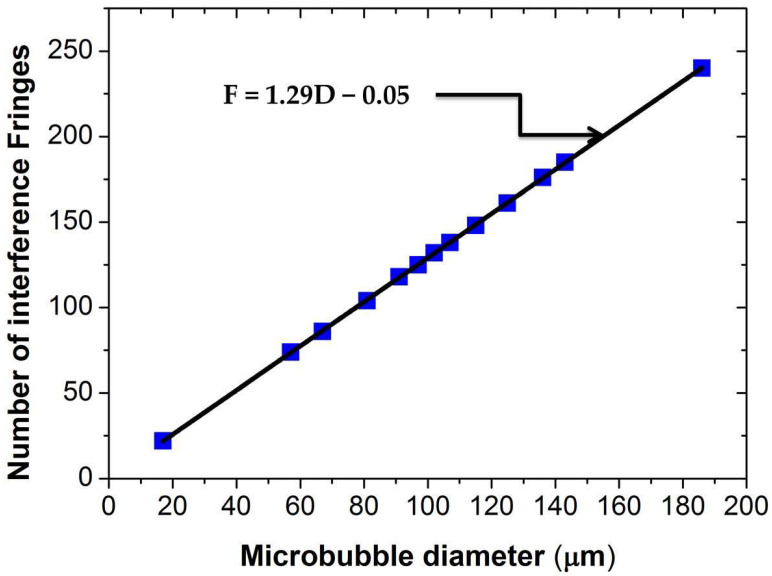
Relationship between the number of interference fringes and the bubble’s diameter.

Figure 6 shows the interference fringes as a function of the microbubble’s size, in which can be seen the relationship lineal between the number of interference fringes (NIF) and the bubble’s diameter. Here, the NIF tended to increase linearly when the bubble’s diameter increased, which indicates a perfect association between both variables. It should be mentioned that, when the microbubble growth rate is slower than 4.75 mm/s, the interference fringes cannot be visualized in the oscilloscope, as mentioned above.

## 5. Application

A microbubble is a spherical shell which contains gas; therefore, microbubbles can be used as pressure probes because their size changes with pressure changes [24,25]. When the pressure pulses produced by a loudspeaker pass through the microbubble, they provoke a contraction and expansion in its volume. A positive pressure change corresponds to a compression of the bubble, while a negative pressure change is related to the expansion of it. Therefore, the surface S2 will be oscillating, changing the length of the cavity, *l*. The frequency of vibration is $f_{vib}=1/T$, where *T* is the time period of a “full cycle”.

**Figure 7:**
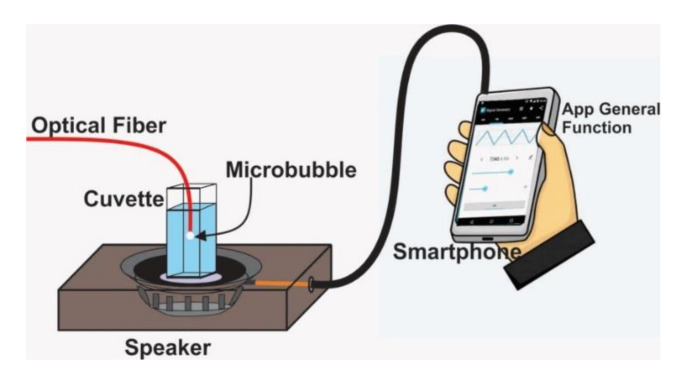
Experimental setup of a vibration sensor based in Doppler interferometry.

As a demonstration of a practical application, we used a microbubble induced at the tip of the optical fiber as a sensor to detect and quantify the oscillation frequency of the cuvette (with ethanol), in which the bubble is produced (Figure 7). For this demonstration, zinc nanoparticles were photodeposited on the tip of the optical fiber. In this case, these nanoparticles caused a loss of 4 dB, and the microbubble was generated with an optical power of 15 mW. We placed the cuvette on a portable speaker (mps-70 from Sony) to generate vibrations on it, as can be observed in Figure 7. In order to generate the microbubble on the optical fiber end and measure the oscillation frequency of the cuvette, the same experimental setup shown in Figure 2 was used. A sinusoidal wave was sent to the speaker through a smartphone, to which an application was installed to generate waveforms. Once the microbubble reaches a size of 83 or 175 µm, the laser power is reduced to 5 mW, in order to reduce (or even stop) the growth velocity so that the microbubble increases its size slowly and the measurements can be compared. If the laser power is not reduced, the microbubble will continue growing, and when the cuvette is subjected to vibrations, two types of interference fringes will be observed in the oscilloscope. The first is due to the growth of the bubble, and the second is due to the vibration of the cell.

**Figure 8:**
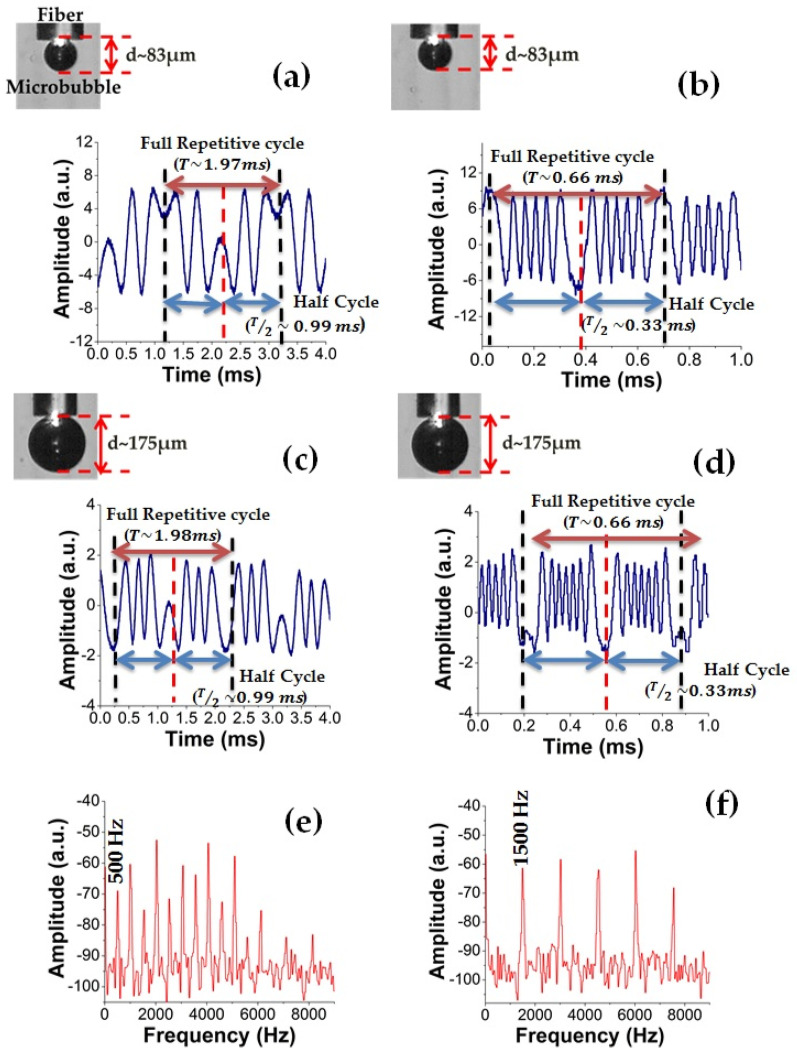
Real time of the vibration sensor using a microbubble with a diameter of (**a**,**b**) 83 µm and (**c**,**d**) 175 µm. (**e**,**f**) Fast Fourier Transform obtained with the oscilloscope from signals shown in (**a**,**b**), respectively.

Figure 8 shows the response of vibration sensor obtained with the oscilloscope when a sinusoidal wave at a frequency of 500 and 1500 Hz was sent to the speaker. Microbubbles formed on the tip of the optical fiber end with sizes of 83 and 175 µm were used as Fabry–Perot cavities. It is important to note that the frequency of vibration (*f_vib_* = 1/*T*) obtained from this figure agrees with the signal sent to the speaker through the smartphone. Furthermore, the vibration frequency was verified through the Fast Fourier Transforms (FFT), which was obtained by using the oscilloscope (Figure 8e,f). The fundamental frequency coincides as expected with the frequency obtained from Figure 8a,b. It is possible to observe that the number of interference fringes increases with the oscillation frequency. For instance, when the microbubble had a size of 83 µm, the interference fringes observed were two and five, considering an oscillation frequency of 500 and 1500 Hz, respectively. Moreover, for a microbubble whose size was approximately 175 µm, the interference fringes observed were three and seven, considering an oscillation frequency of 500 and 1500 Hz, respectively. Although in our experiments the vibration was determined by the mechanical response of the overall system, the frequency response of the measuring technique was limited by the response time of the detector, as it was mentioned previously. It is important to mention that vibrations with frequencies greater than 2 kHz were not detected, because the oscillation of the liquid–vapor interface is so small that the signal is below the noise floor of the photodetector; therefore, the interference fringes were not displayed in the oscilloscope. A photodetector with smaller bandwidth will improve the performance. Furthermore, interference fringes were not observed when the microbubble was greater than 200 µm. We believe that this is due to the loss in the optical power.

Finally, we must point out that there are two types of interference fringes: The first are those caused by the growth of the microbubble due to heating of nanoparticles by the absorption radiation (see Figure 3), and the second is due to the vibration of the cuvette (see Figure 7). Therefore, when we observe in the oscilloscope the seconds interference fringes, these are modulated amplitude by the first interference fringes generating frequency breakdown points. It is worth mentioning that it is possible to apply the wavelet transform to identify the frequency breakdown points of the signal efficiently and automatically count the interference fringes; however, this is not part of the purpose of this work.

## 6. Conclusions

In the present work, the experimental measurement of the growth of a microbubble induced by ZnNPs photodeposited on the tip of an optical fiber was reported. The microbubble was studied by analyzing it as an Fabry–Perot cavity, where its size was quantified in the time domain through the number of interference fringes, and then the results were verified by obtaining images by a high-speed camera. Furthermore, we realized a demonstration by using the microbubble as a vibration sensor, in which it was possible to know the vibration frequency of a cuvette. The study presented in this work shows several advantages over conventional methods, such as the low cost, real-time measurement, and simplification in the experimental arrangement, because the laser source is the same to the generation of the bubble and to the analysis of the evolution of the growth of a microbubble. This method can be used for the characterization of the dynamics of a microbubble in continuous growth induced at the end of an optical fiber, opening the possibility to future applications for the sensing of pressure, strain, and temperature, etc.

## Data Availability

Some or all date generated or used during the study are available from corresponding author by request.
